# Elevated CO_2_ Influences Nematode-Induced Defense Responses of Tomato Genotypes Differing in the JA Pathway

**DOI:** 10.1371/journal.pone.0019751

**Published:** 2011-05-24

**Authors:** Yucheng Sun, Jin Yin, Haifeng Cao, Chuanyou Li, Le Kang, Feng Ge

**Affiliations:** 1 State Key Laboratory of Integrated Management of Pest and Rodents, Institute of Zoology, Chinese Academy of Sciences, Beijing, People's Republic of China; 2 State Key Laboratory of Plant Genomics, National Centre for Plant Gene Research, Institute of Genetics and Developmental Biology, Chinese Academy of Sciences, Beijing, People's Republic of China; The George Washington University, United States of America

## Abstract

Rising atmospheric CO_2_ concentrations can affect the induced defense of plants against chewing herbivores but little is known about whether elevated CO_2_ can change the induced defense of plants against parasitic nematodes. This study examined the interactions between the root-knot nematode *Meloidogyne incognita* and three isogenic tomato (*Lycopersicon esculentum*) genotypes grown under ambient (390 ppm) and elevated (750 ppm) CO_2_ in growth chambers. In a previous study with open-top chambers in the field, we reported that elevated CO_2_ increased the number of nematode-induced root galls in a JA-defense-dominated genotype but not in a wild-type or JA-defense-recessive genotype. In the current study, we tested the hypothesis that elevated CO_2_ will favor the salicylic acid (SA)-pathway defense but repress the jasmonic acid (JA)-pathway defense of plants against plant-parasitic nematodes. Our data showed that elevated CO_2_ reduced the JA-pathway defense against *M. incognita* in the wild-type and in a genotype in which defense is dominated by the JA pathway (a JA-defense-dominated genotype) but up-regulated the SA-pathway defense in the wild type and in a JA-defense-recessive genotype (jasmonate-deficient mutant). Our results suggest that, in terms of defense genes, secondary metabolites, and volatile organic compounds, induced defense of nematode-infected plants could be affected by elevated CO_2_, and that CO_2_-induced changes of plant resistance may lead to genotype-specific responses of plants to nematodes under elevated CO_2_. The changes in resistance against nematodes, however, were small relative to those reported for chewing insects.

## Introduction

Global atmospheric CO_2_ concentration has increased by approximately 40% from a pre-industrial value of 280 ppm to 387 ppm in 2009, and is anticipated to double by the end of this century [1]. Along with its direct effect on plant physiology and growth, elevated CO_2_ typically reduces the quality of plants by increasing the C:N ratio, and causes plants to re-allocate assimilates to the synthesize secondary metabolites, thereby altering the interactions between host plants and herbivores [2], [3].

Generally, elevated CO_2_ does not trigger or alter the induced-defense processes of undamaged plants but may modify the induced defense of plants damaged by herbivores [4], [5]. For example, elevated CO_2_ increased the susceptibility of soybean to Japanese beetle and western corn rootworm by down-regulating the expression of genes related to the jasmonic acid (JA) pathway [6], [7]. Although elevated CO_2_ impairs the JA defense response against these chewing insects, the effect of elevated CO_2_ on defense responses induced by plant-parasitic nematodes (i.e., JA, salicylic acid, antioxidant) has not been investigated.

The root-knot nematode *Meloidogyne incognita* is an obligate endoparasite that feeds exclusively on the cytoplasm of living plant cells [8]. *M. incognita* infects a large number of crops and causes severe losses in yield. The disease symptoms on infected plants include galls on the roots, stunted growth, wilting, and increased susceptibility to other pathogens [9]. Effects of elevated CO_2_ on nematode densities as mediated by the host plant are “plant species-specific” and include negative effects [10], positive effects [11], and no significant effects [12]. Most of these studies proposed that changes in root biomass and C/N ratio were the main factors responsible for the effects of elevated CO_2_ on nematode abundance [10]–[12]. However, the mechanisms underlying the effect of elevated CO_2_ on the interaction between plant-parasitic nematodes and their host plants are poorly understood.

Systemic acquired resistance (SAR) is considered to be the major induced plant defense that confers long-lasting protection against nematodes [13]. SAR depends on the salicylic acid (SA) pathway and is associated with accumulation of pathogenesis-related proteins, which are considered to contribute to resistance. Researchers have recently suggested, however, that the JA pathway is also an indispensable component of plant resistance to nematodes [14], [15]. The JA pathway is associated with expression of proteins (including proteinase inhibitors, phenylalanine ammonialyase, and lipoxygenase), up-regulation of secondary metabolites, and induction of plant volatile organic compounds (VOC). Cooper *et al.* (2005) reported that the artificial induction of JA-pathway defenses reduced reproduction of root-knot nematodes on tomato plants [16]. Our previous research also found that a tomato genotype (*35S::Prosystemin*) in which induced defense was dominated by the jasmonic acid (JA) pathway (hereafter referred to as a “JA defense-dominated genotype”) has stronger resistance to nematodes than a JA defense-recessive genotype (*spr2*, a jasmonate-deficient mutant) and that the specific responses of these isogenic tomato genotypes to elevated CO_2_ requires our further investigation [17].

Based on several works referring to plant induced defense under elevated CO_2_ and our previous work [6], [7], [17], we hypothesized that elevated CO_2_ would reduce the resistance of a JA defense-dominated genotype against *M. incognita* by altering the JA pathway but enhance the SA-pathway defense of a JA-defense-recessive genotype infected by *M. incognita*. In this study, we determined whether elevated CO_2_ affects the regulation of genes and the production of secondary metabolites and the emission of VOC associated with the JA pathway of isogenic tomato genotypes. We also determined whether elevated CO_2_ affects the regulation of genes associated with the SA pathway. Finally, we determined whether the changes in these pathways and genes are associated with the performance of *M*. *incognita* under elevated CO_2_.

## Results

### Temporal gene expression in leaves

**Figure 1:**
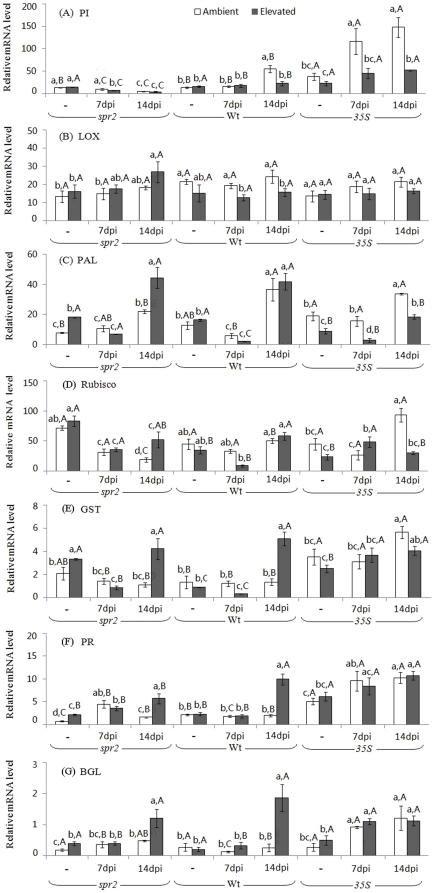
Expression levels of seven target genes of tomato genotypes grown under ambient and elevated CO_2_ without (“-”) and with *M. incognita*; plants with *M. incognita* were sampled 7 days post-inoculation (7 dpi) and 14 dpi. Each value represents the average (±SE) of four replicates. Different lowercase letters indicate significant differences among combinations of nematode and CO_2_ level within the same tomato genotype (LSD test: d.f. = 5, 18; *P<*0.05). Different uppercase letters indicate significant differences among tomato genotypes within the same CO_2_ and nematode treatment (LSD test: d.f. = 2, 9; *P<*0.05).

Elevated CO_2_ increased *PAL*, *GST*, *PR1*, and *BGL2* levels of uninfected *spr2* plants (Figure 1, Table S1). In contrast, elevated CO_2_ reduced the *PAL* level only of uninfected *35S* plants (F_1,6_ = 9.16, *P* = 0.023). Regardless of CO_2_ level, uninfected *35S* plants had the highest *PI1* and *PR1* levels among the genotypes. Furthermore, regardless of CO_2_ level, the 14-dpi treatment (nematodes added 14 days before sampling) increased *PAL*, *PR1*, and *BGL2* levels and reduced the *PI1* and *RUBISCO* level of *spr2* plants, and increased the *PI1*, *PAL*, *GST*, *PR1*, and *BGL2* levels of *35S* plants. The 14-dpi treatment increased the *PI1* and *PAL* levels of Wt plants under ambient CO_2_ and the *GST*, *PR1*, and *BGL2* levels under elevated CO_2_. Elevated CO_2_ reduced the *PI1* and *LOX* levels of Wt plants but increased the *GST*, *PR1*, and *BGL2* levels of *spr2* and Wt plants at 14-dpi. Elevated CO_2_ decreased the *PI1* and *PAL* levels of *35S* plants at 7- and 14-dpi (Figure 1).

### Levels of proteins, amino acids, and secondary metabolites

**Figure 2:**
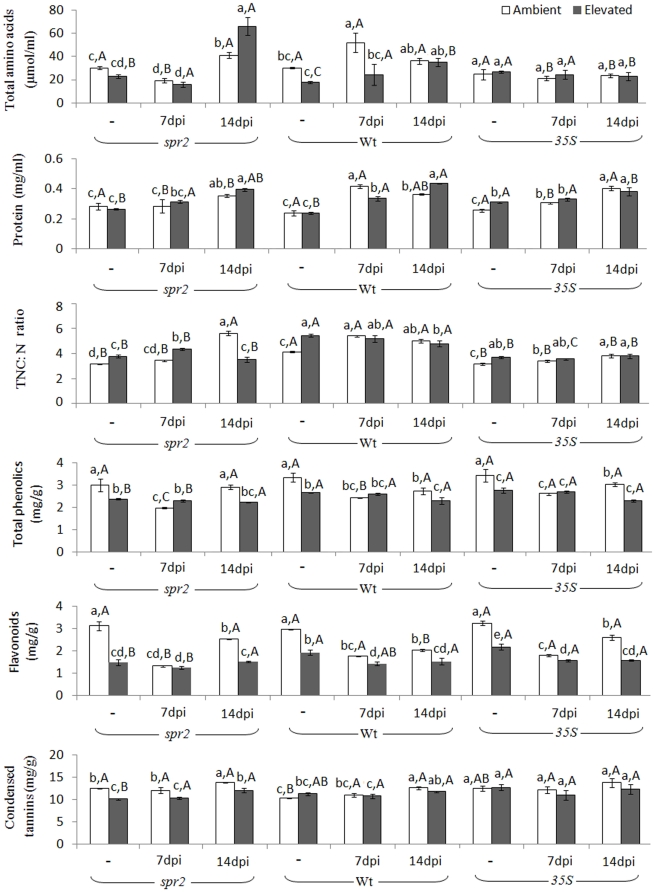
Foliar chemical components of tomato genotypes grown under ambient and elevated CO_2_ without (“-”) and with *M. incognita*; plants with *M. incognita* were sampled 7 days post-inoculation (7 dpi) and 14 dpi. Each value represents the average (±SE) of three replicates. Different lowercase letters indicate significant differences among combinations of nematode and CO_2_ level within the same tomato genotype (LSD test: d.f. = 5, 12; *P<*0.05). Different uppercase letters indicate significant differences among tomato genotypes within the same CO_2_ and nematode treatment (LSD test: d.f. = 2, 6; *P<*0.05).

Elevated CO_2_ increased the protein level of *35S* plants and the foliar TNC:N ratio of all three genotypes. In contrast, elevated CO_2_ reduced the total phenolics and flavonoids of all the genotypes and the condensed tannins level of *spr2* plants (Figure 2, Table S2). Regardless of CO_2_ level, uninfected Wt plants had the highest foliar TNC:N ratio among the genotypes. Furthermore, regardless of CO_2_ level, the 7-dpi treatment reduced whereas the 14-dpi treatment increased amino acid level of *spr2* plants. The 14-dpi treatment increased the protein level of all the genotypes and the TNC:N ratio of all the genotypes under ambient CO_2_. Regardless of CO_2_ level, the 14-dpi treatment reduced total phenolics and flavonoids of Wt plants and flavonoids of *35S* plants but increased condensed tannins of *spr2* plants (Figure 2).

### Volatile emission rate

**Figure 3:**
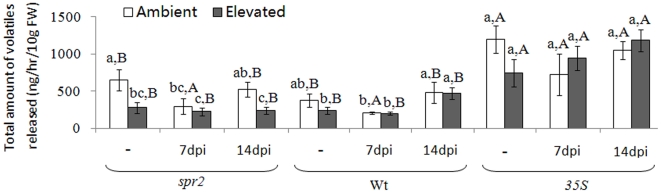
Emission rate of total volatile organic compounds (VOC) from tomato genotypes grown under ambient and elevated CO_2_ without (“-”) and with *M. incognita*; plants with *M. incognita* were sampled 7 days post-inoculation (7 dpi) and 14 dpi. Each value represents the average (±SE) of three replicates. Different lowercase letters indicate significant differences among combinations of nematode and CO_2_ level within the same tomato genotype (LSD test: d.f. = 5, 12; *P<*0.05); Different uppercase letters indicate significant differences among tomato genotypes within the same CO_2_ and nematode treatment (LSD test: d.f. = 2, 6; *P<*0.05). Emission rate represents ng of compound released by 10 g (fresh weight) of leaves per hour.

CO_2_ level, tomato genotype, nematode infection, and the interaction between CO_2_ and nematode significantly affected the total amount of VOC (Table S3). In the absence of nematodes, elevated CO_2_ reduced the total amount of VOC released by *spr2* plants. The jasmonate-deficient *spr2* and Wt plants released less VOC than *35S* plants under both ambient and elevated CO_2_ (Figure 3). Elevated CO_2_ reduced emission of ocimene and *β*-phellandrene in uninfected *spr2* plants and hexenal in uninfected *35S* plants. In the 14-dpi treatment under elevated CO_2_, *spr2* plants emitted less of each volatile terpene than *35S* plants (Table S4).

### Galls resulting from nematode infection

**Figure 4:**
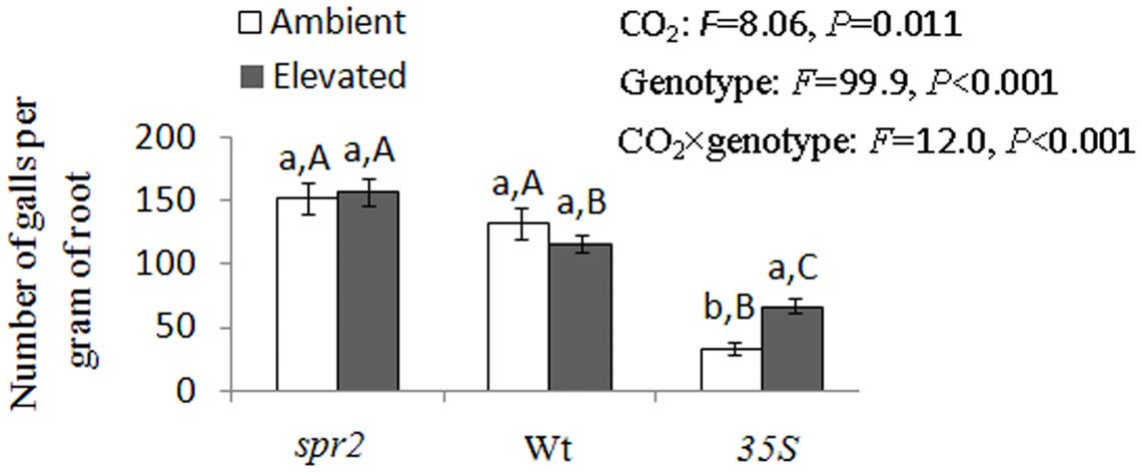
Number of galls per gram of dry root infected by *M. incognita* 14 days post-inoculation on tomato genotypes grown under ambient (390 ppm) and elevated CO_2_ (750 ppm). Each value represents the average (±SE) of four replicates. Different lowercase letters indicate significant differences between CO_2_ levels within the same tomato genotype (LSD test: df = 1,16, *P *<0.05); different uppercase letters indicate significant differences among tomato genotypes within CO_2_ levels (LSD test: d.f. = 2,24, *P *<0.05).

CO_2_ level, tomato genotype, and their interactions affected the number of nematode-induced galls per gram of dry root (Figure 4). The number of galls on *35S* roots was greater under elevated CO_2_ than under ambient CO_2_ (F_1,16_ = 78.3, *P*<0.001). Regardless of CO_2_ level, there were fewer galls on *35S* plants than on Wt or *spr2* plants (Figure 4). Under elevated CO_2_, galls were more abundant on *spr2* roots than on the roots of the other two genotypes (F_2,24_ = 50.7, *P*<0.001).

## Discussion

Although numerous studies have focused on the evolution of inducible defenses against herbivory, much less emphasis has been placed on how CO_2_ and other aspects of the abiotic environment affect these inducible responses [18]. Bidart-Bouzat *et al.* (2005) reported that elevated CO_2_ increased induced defense (i.e., increased glucosinolate levels) of *Arabidopsis thaliana* against diamondback moths [19]. Moreover, Zavala *et al.* (2009) showed that elevated CO_2_ down-regulated the gene expression and activity of cysteine proteinase inhibitors, which are the principal defenses of soybean against insect herbivores [7]. An interaction between CO_2_ level and herbivory, however, has seldom been detected in host plants [20]. Our results show that in the jasmonate-deficient mutant *spr2*, elevated CO_2_ up-regulated the induced defense at 14-dpi based on the SA pathway, including *PR1* and *BGL2* genes, but did not up-regulate the induced defense based on the JA pathway. Conversely, in the JA defense-dominated genotype *35S*, elevated CO_2_ decreased induced defense based on the JA pathway (i.e., CO_2_ reduced the *PI1* level) but did not increase induced defense at 7- and 14-dpi based on the SA pathway. Thus, our results support the hypothesis that, under elevated CO_2_, JA defense-dominated genotypes tend to express reduced JA-pathway-induced defense and JA defense-recessive genotypes tend to amplify the SA-signaling pathway. To the best of our knowledge, this is the first study demonstrating that nematode-induced defense based on the JA or SA pathway in plants can be modified by CO_2_ level (significant CO_2_×nematode interactions for *PI1* and *PR1* genes, Table S1), and that these changes can differ among three isogenic tomato genotypes (significant CO_2_×nematode×genotype interaction).

Increased tissue levels of reactive oxygen species like H_2_O_2_ and O_2_^−^ and the metabolism of glutathione induced by nematode infection are linked to defensive/secondary metabolism and cell differentiation of plant roots [21]. The results presented in this study show that, under ambient CO_2_, nematode infection up-regulated the *GST* gene in leaves only in the JA defense-dominated genotype *35S* and that elevated CO_2_ increased levels of the *GST* gene in *spr2* and Wt plants infected by nematodes at 14-dpi. Furthermore, for all three genotypes, nematode infection up-regulated the foliar *PAL* gene but only increased condensed tannins levels. Regardless of nematode infection, elevated CO_2_ decreased the level of the *PAL* gene only in *35S* plants but unexpectedly reduced levels of total phenolics and flavonoids in all three genotypes. These results can probably be explained by elevated CO_2_ inhibiting the activity of the *PAL* enzyme at the post-transcriptional level rather than at the transcriptional level. Interestingly, the results of research investigating the effect of elevated CO_2_ on plant volatiles emissions are controversial [22], [23]. Our finding of reduced VOC (including monoterpenes and sesquiterpenes) emissions from *spr2* plants is consistent with the results of Loreto *et al.* [22], who demonstrated that, because of down-regulation of terpene synthase activity, elevated CO_2_ reduced emission of monoterpenes from *Quercus ilex* leaves by approximately 68%. The authors suggested that VOC is probably limited by the availability of photosynthetic carbon. In addition, Vuorinen *et al.* (2004) also reported that elevated CO_2_ decreased the emission of JA-regulated terpene volatiles in cabbage [24].

In accordance with previous studies and because of photosynthetic acclimation, down-regulation of the mRNA level of the *RUBISCO* gene in Wt plants was observed under elevated CO_2_ (Figure 1D). Furthermore, as predicted by the Carbon Nutrient Balance (CNB) hypothesis [25], accumulated ‘extra’ carbon (relative to nitrogen) increases the plant C/N ratio and consequently would increase carbon-based defenses. Our data, however, showed that most carbon-based secondary metabolites including total phenolics, flavonoids, monoterpenes, and sesquiterpenes volatiles were decreased by elevated CO_2_. It seems likely that the ‘extra’ carbon might be allocated to the formation of other secondary metabolites and that the response of different biosynthetic pathways to elevated CO_2_ could be species-specific or dependent on the developmental stage of plants. Moreover, the induction of a higher C/N ratio and lower nitrogen content by elevated CO_2_ often results in lower plant quality (i.e., reduced quantities of amino acids and protein) [26]. In contrast, our relatively short-term study found no evidence that elevated CO_2_ reduced the levels of amino acids and protein, which is not consistent with results from our previous, relatively long-term study in open-top chambers (OTC) in the field [17]. Perhaps long-term cumulative effects of elevated CO_2_ contributed to these differences.

In plants, JA and SA are ubiquitous signals of induced resistance against many if not most herbivores and pathogens, respectively [14]. Although the widespread acceptance that the accumulation of SA is the major signaling pathway of plant response to nematode infection [27], JA has been recently proven to be another efficient plant defense against nematodes [28]. For example, Cooper *et al.* (2005) reported that foliar application of JA suppressed the reproduction of the nematode *Melodoigyne javanica* on tomatoes [16]. In our current growth chamber study and in our previous field OTC study, a JA defense-dominated genotype exhibited higher resistance to nematodes than wild types or JA defense-recessive genotypes [17]. Typically, nematode infection primarily induces SA-mediated defense responses because nematode infection generates only minor trauma. Our data show that, under ambient CO_2_ level, nematode infection triggers the SA pathway at 14-dpi and involves the up-regulation of *PR1* and *BGL2* genes in jasmonate-deficient *spr2* mutants but not in Wt plants. These results suggest that induction of SA-mediated defense did not confer resistance to nematodes. In contrast, *35S* transgenic plants over-express prosystemin, which can constitutively activate the JA pathway in unwounded plants and result in stronger and quicker induced resistance in response to damage by herbivores. Nematode infection consequently triggered the up-regulation of the JA pathway (*PI1*) and the SA pathway (*PR1* and *BGL2*) at 14-dpi in *35S* plants. Likewise, we found that, regardless of CO_2_ level, the JA defense-dominated genotype *35S* plants had the strongest resistance to nematodes among three genotypes. Accordingly, nematodes may tend to activate the ineffective defense pathway (SA-mediated defense) but suppress effective induced defense (JA-mediated defense) of plants.

Elevated CO_2_ tends to affect plant hormones and thereby could modify the induced defense against nematodes. Li *et al.* (2002b) demonstrated that elevated CO_2_ sharply increased the levels of several plant hormones including indole-3-acetic acid, gibberellins, isopentenyladenosine, and zeatin riboside [29]. Additionally, our data show that, at 14-dpi, elevated CO_2_ enhances the induced defense based on the SA pathway in *spr2* and Wt plants but suppressed the induced defense based on the JA pathway in *35S* and Wt plants. This is probably because elevated CO_2_ affects plant defense responses through pathway cross-talk, amplifying the SA-signaling pathway to repress the JA-signaling pathway in Wt plants. Thus, an explanation of our results may be that elevated CO_2_ repressed the JA-signaling pathway but did not trigger the defense based on the SA pathway in *35S* plants, making the *35S* plants more susceptible to nematodes under elevated CO_2_ level.

Elevated CO_2_ down-regulates the gene expression of JA-dependent pathway defense, reduces the activity of PI, and in turn increases the susceptibility of soybean to both below-ground and above-ground chewing insects [6]. In contrast, although our study indicated that elevated CO_2_ had a genotype-specific effect on JA and SA-dependent pathway defense, the nematode resistances in the wild type and in the JA-defense-recessive genotype were not changed by elevated CO_2_, and the JA-defense-dominated genotype still had the strongest nematode resistance among genotypes under elevated CO_2_. Thus, the CO_2_-induced changes in systemic resistance against parasitic nematodes were substantially smaller than those reported for chewing insects.

In conclusion, this study demonstrates that, as indicated by secondary metabolites, VOC, and genes associated with defense against herbivores, induced defense of nematode-infected plants could be affected by global CO_2_ changes, and that CO_2_-induced changes in plant resistance may lead to genotype-specific changes in the response of plants to nematodes under elevated CO_2_. Theory predicts that plant defenses are costly [30], [31]. In this respect, our work provides a foundation for further research on how elevated CO_2_ affects the tradeoff between resistance and tolerance of different isogenic genotypes. Finally, future research on the effects of elevated CO_2_ on induced-defense responses of plants should consider multiple signaling pathways.

## Materials and Methods

### Atmospheric CO_2_ concentration treatments

This experiment was performed in six closed-dynamic CO_2_ chambers (CDCC; Safe PRX-450B, 68 cm long, 68 cm wide and 185 cm high) [32]. The chambers were maintained at 26±0.8°C, 70±2% RH, and 14∶10 (L:D)-h photoperiod with 12,000 LX of active radiation supplied by 18 fluorescent lamps (60-W) in each chamber.

Two CO_2_ levels, 390±30 ppm (current ambient level) and 750±30 ppm (predicted level at the end of this century), were applied. Three chambers were used for each CO_2_ treatment. Elevated CO_2_ concentrations were monitored and adjusted with an infrared CO_2_ transmitter (Ventostat 8102, Telaire Company, Goleta, CA, USA) once every minute to maintain relatively stable CO_2_ concentrations. The automatic-control system for maintaining CO_2_ levels was described in detail by Chen and Ge [32].

### Host plants and nematodes

Wild-type (Wt) tomato plants (*L. esculentum* cv. Castlemart), the jasmonate-deficient *spr2* mutants (*spr2*), and the *35S::Prosystemin* transgenic tomato plants (*35S*) were kindly provided by Professor C. Li of the Institute of Genetics and Developmental Biology, the Chinese Academy of Sciences. The JA-biosynthesis mutant, *suppressor of prosystemin-mediated responses2* (*spr2*), reduces chloroplast ω3 fatty acid desaturase, which impairs the synthesis of JA [33]. In contrast, *35S::prosystemin* (*35S*) transgenic plants over-express prosystemin, which constitutively activates systemic defense in unwounded plants and results in stronger and quicker induced resistance [34]. Tomato (*L. esculentum*) cv Castlemart was the Wt parent for both the *spr2* mutant and the *35S* transgenic genotypes. Tomato seeds of the three genotypes were sown individually in plastic pots (15 cm diameter and 16 cm high) filled with 4∶1 (v/v) sterilized loamy soil:earthworm feces. Tomato plants were exposed to the CO_2_ treatments after seedling emergence, and plants were randomly repositioned within each chamber weekly to minimize position effects. No chemical fertilizers and insecticides were used. Water was added to each pot once every 2 days.

The root-knot nematode, *M. incognita*, was cultured on Wt plants grown under ambient CO_2_. To prepare nematode inoculum, nematode eggs were extracted from infected tomato roots by blending them in water containing 10% bleach (CaCl_2_·Ca(OCl)_2_·2H_2_O). Eggs and root debris were collected on a 25-µm-pore sieve. The second-stage juveniles (J2) were hatched from the eggs and used as inoculum [35].

After plants had grown in the CDCCs for 4 weeks, 6 plants of each genotype in each CDCC were randomly selected ( = 18 plants per CDCC and 108 plants in total) and inoculated with freshly hatched *M. incognita* J2, and another 6 plants of each tomato genotypes in each CDCC were treated with water as the control. One week later, 6 additional plants of each tomato genotype in each CDCC were inoculated with *M. incognita* as described above. All the nematode-treated pots received ≈3000 J2 in 5 ml of water applied with a pipette over the surface of the soil around the primary roots. One week after the second inoculation, the experiment was terminated and tomato plants were sampled. Thus, the experiment had two levels of CO_2_, three tomato genotypes, and three nematode levels (uninfected control, nematodes added 7 days before sampling, and nematodes added 14 days before sampling). The latter two nematode treatments are referred to as the 7- and 14-dpi (days post inoculation) treatments, and the uninfected plants are referred to as controls.

### Assessment of plant traits and foliar chemical components

Three plants from each combination of tomato genotype and nematode treatment in each chamber ( = 27 plants per CDCC and 162 plants in total) were randomly selected. Leaves and roots from each plant were collected and stored at −20°C until subjected to chemical analysis, except that a sample of fresh leaves (0.5 g) from each plant was removed and stored at −70°C for real-time PCR, as described later in this subsection.

The chemical components of the tomato leaves were analyzed. Protein concentrations were determined by the Bradford (1976) assay [36]. Total amino acids (TAA) were analyzed with a reagent kit (Nanjing Jiancheng Bioengineering Institute, Nanjing, Jiangsu Province, China) [37]. Total non-structural carbohydrates (TNCs), mainly starch and sugar, were assayed by acid hydrolysis following the method of Tissue and Wright (1995) [38]. Nitrogen content was assayed using Kjeltec nitrogen analysis (Foss automated Kjeltec™ instruments, Model 2100). Total phenolics were analyzed by the Folin-Ciocalteu method described by Kujala *et al.* (2000) [39]. Flavonoids and condensed tannins were measured using the methods of Jia *et al.* (1999) and Terrill *et al.* (1992), respectively [40], [41].

### Real-time quantitative PCR

Each treatment combination was replicated four times for biological repeats, and each biological repeat contained three technical repeats. The RNeasys Mini Kit (Qiagen) was used to isolate total RNAs from tomato leaves (0.5 g from samples stored at −70°C; see the first paragraph of the previous subsection), and 2-µg quantities of the RNAs were used to generate the cDNAs. The mRNA amounts of 7 target genes were quantified by real-time quantitative PCR; the target genes were proteinase inhibitor (*PI1*), lipoxygenase (*LOX*), ribulose-1, 5-bisphosphate carboxylase/oxygenase (*RUBISCO*), phenylalanine ammonia lyase (*PAL*), glutathione-S-transferase (*GST*), pathogenesis-related protein (*PR1*), and β-1,3-glucanase (*BGL2*). Specific primers for each gene selected were designed from the tomato EST sequences using PRIMER5 software (Table S5). The PCR reactions were performed in a 20-µL total reaction volume including 10 µL of 2×SYBRs Premix EX TaqTM (Qiagen) master mix, 5 mM each of gene-specific primers, and 1 µL of cDNA templates. They were carried out on the Mx 3000P detection system (Stratagene), and the parameters were as follows: 2 min at 94°C; then 40 cycles of 20 s at 95°C, 30 s at 56°C, and 20 s at 68°C; and finally one cycle of 30 s at 95°C, 30 s at 56°C, and 30 s at 95°C. This PCR protocol produced the melting curves, which can be used to judge the specificity of PCR products. A standard curve was derived from the serial dilutions to quantify the copy numbers of target mRNAs. The relative level of each target gene was standardized by comparing the copy numbers of target mRNA with *β*-actin (the house keeping gene) copy numbers, which remain constant under different treatment conditions. The *β*-actin mRNAs of the control were examined in every PCR plate to eliminate the systematic error.

### Collection and quantification of plant volatiles

Volatiles were collected from one randomly selected plant from each combination of tomato genotype and nematode treatment in each chamber ( = 9 plants per CDCC and 54 plants in total). The headspace volatiles were collected according to Turlings *et al.* (1998) [42]. The shoots and leaves of each plant, except for the stem extending 4 to 5 cm from the soil surface, were sealed in a plastic bag (40 cm wide and 46 cm long). Purified air was pumped (Beijing Institute of Labor Instruments, China) into the bag through a freshly activated charcoal trap (Beijing Chemical Company) and then withdrawn through a glass cartridge (3.0 mm internal diameter and 12.6 cm long) packed with 100 mg of the adsorbent Porapak Q (80–100 mesh, Supelco, Bellefonte, PA, USA); the flow rate was 0.25 L/min. Volatile compounds were rinsed from the Porapak Q with 600 µl of *n*-hexane (HPLC grade, Sigma-Aldrich, USA) containing internal standards (200 ng of ethyl heptanoate) for quantification. The aeration extracts were stored at −20°C until analyzed. Immediately after headspace volatiles were collected, the fresh weights of the plant leaves were measured.

Volatiles were quantified and identified using a gas chromatography-mass spectrometry (GC-MS) system (Hewlett Packard 6890N GC model coupled with 5973 MSD) equipped with a HP-5MS column (60 m long, 0.25 mm inner diameter, and 0.25 µm film thickness; Agilent Technologies, Palo Alto, CA, USA). The initial oven temperature was kept at 50°C for 1 min and then increased to 250°C at a rate of 5 C°/min. Volatile compounds were identified by comparing their retention times and spectra with those of compounds in the NIST02 library (Scientific Instrument Services, Inc., Ringoes, NJ, USA) and those of pure standards.

### Assessment of disease symptoms caused by the nematode

When J2 of *M. incognita* infect roots, galls may occur, and galls were quantified to estimate root infection. Roots of 14-dpi nematode infected plants (3 plants from each combination of tomato genotype and nematode treatment in each chamber, 27 plants per CDCC and 162 plants in total) were carefully removed from soil and washed. A stereomicroscope was used to count the number of galls produced on the entire root system of each plant.

### Statistical analyses

Statistical analyses were performed with SAS software (SAS Institute, 2002). Differences in gene regulation, foliar chemical components, and VOC were analyzed by MANOVA (ANOVA, SAS Institute, 1996). The number of root galls on different tomato genotypes under two CO_2_ levels was assessed with a two-way ANOVA. Least significant difference (LSD) tests were used to separate the levels within the same variable. Proportional data were subjected to arcsine square root transformation. Other data were square root (X), ln(X), or arcsin(X) transformed to satisfy assumptions of normality if necessary.

## Supporting Information

Table S1 *P* values from MANOVAs for the effect of CO_2_ level, tomato genotype, and nematode infection on the relative mRNA level of genes involved in plant defense and photosynthesis.
(DOC)

Table S2 *P* values from MANOVAs for the effect of CO_2_ level, tomato genotype, and nematode infection on foliar chemical components of three tomato genotypes.
(DOC)

Table S3 *P* values from MANOVAs for the effect of CO_2_ level, tomato genotype, and nematode infection on plant volatiles.
(DOC)

Table S4 Emission rate^a^ of volatile organic compounds (VOC) from tomato genotypes grown under ambient (390 ppm) and elevated CO_2_ (750 ppm) without and with *M. incognita*.
(DOC)

Table S5 Primer sequences used in the real-time quantitative PCR.
(DOC)
