# Supplementary material for: Elevated CO2 Influences Nematode-Induced Defense Responses of Tomato Genotypes Differing in the JA Pathway
Source: PLoS One. 2011 May 24;6(5):e19751. doi: 10.1371/journal.pone.0019751 (PMC3101209; doi:10.1371/journal.pone.0019751)
Supplement: Table S1 — P values from MANOVAs for the effect of CO2 level, tomato genotype, and nematode infection on the relative mRNA level of genes involved in plant defense and photosynthesis. (DOC) [file pone.0019751.s001.doc]

***Table S1.***  *P values from MANOVAs for the effect of CO_2_ level, tomato genotype, and nematode infection on the relative mRNA level of genes involved in plant defense and photosynthesis.*

|  | Main effects and interactions | | | | | | |
| --- | --- | --- | --- | --- | --- | --- | --- |
| Gene mRNA (dependent variable) | CO_2_^a^ | Genotype^b^ | Nematode^c^ | CO_2_×Genotype | CO_2_×Nematode | Genotype×Nematode | CO_2_×Genotype×Nematode |
| *PI* | <0.001*** | <0.001*** | <0.001*** | <0.001*** | <0.001*** | <0.001*** | <0.001*** |
| *LOX* | 0.836 | 0.450 | 0.018* | 0.004** | 0.606 | 0.601 | 0.762 |
| *PAL* | <0.001*** | 0.608 | <0.001*** | <0.001*** | 0.011 * | <0.001*** | 0.003 ** |
| *RUBISCO* | 0.100 | 0.034 * | <0.001*** | <0.001*** | 0.536 | <0.001*** | <0.001*** |
| *GST* | 0.068 | <0.001*** | <0.001*** | 0.007 ** | <0.001*** | <0.001*** | <0.001*** |
| *PR* | <0.001*** | <0.001*** | <0.001*** | 0.040 * | <0.001*** | 0.001 ** | <0.001*** |
| *BGL* | <0.001*** | 0.005 ** | <0.001*** | 0.446 | 0.064 | 0.816 | <0.001*** |
| ^a^ Ambient CO_2_ vs. elevated CO_2_. ^b^ Three genotypes of tomato (*spr2*, Wt, and *35S::prosys*). ^c^ 7 days post-inoculation or 14 days post-inoculation or not inoculated with the root-knot nematode *M. incognita*. *<0.05, **<0.01, ***<0.001. | | | | | | | |
