# Supplementary material for: Elevated CO2 Influences Nematode-Induced Defense Responses of Tomato Genotypes Differing in the JA Pathway
Source: PLoS One. 2011 May 24;6(5):e19751. doi: 10.1371/journal.pone.0019751 (PMC3101209; doi:10.1371/journal.pone.0019751)
Supplement: Table S2 — P values from MANOVAs for the effect of CO2 level, tomato genotype, and nematode infection on foliar chemical components of three tomato genotypes. (DOC) [file pone.0019751.s002.doc]

***Table S2.***  *P values from MANOVAs for the effect of CO_2_ level, tomato genotype, and nematode infection on foliar chemical components of three tomato genotypes.*

|  | Main effects and interactions | | | | | | |
| --- | --- | --- | --- | --- | --- | --- | --- |
| Foliar chemical component (dependent variable) | CO_2_^a^ | Genotype^b^ | Nematode^c^ | CO_2_×Genotype | CO_2_×Nematode | Genotype×Nematode | CO_2_×Genotype×Nematode |
| Protein | 0.132 | 0.005 ** | <0.001*** | 0.445 | 0.092 | <0.001*** | <0.001*** |
| Amino acid | 0.074 | 0.002 ** | <0.001*** | 0.001 ** | 0.006 ** | <0.001*** | 0.024 * |
| TNC:N^4^ | 0.016 * | <0.001*** | <0.001*** | 0.081 | <0.001*** | <0.001*** | <0.001*** |
| Total phenolics | <0.001*** | <0.001*** | <0.001*** | 0.307 | <0.001*** | 0.006 ** | 0.572 |
| Flavonoids | <0.001*** | <0.001*** | <0.001*** | 0.042 * | <0.001*** | 0.002 ** | 0.002 ** |
| Condensed tannins | 0.007 ** | 0.052 | <0.001*** | 0.031 * | 0.255 | 0.836 | 0.364 |
| ^a^ Ambient CO_2_ vs. elevated CO_2_. ^b^ Three genotypes of tomato (*spr2*, Wt, and *35S::prosys*). ^c^ 7 days post-inoculation or 14 days post-inoculation or not inoculated with the root-knot nematode *M. incognita*. ^4^TNC:N ratio represents the total non-structural carbohydrates: total nitrogen ratio. *<0.05, **<0.01, ***<0.001. | | | | | | | |
