# Supplementary material for: Elevated CO2 Influences Nematode-Induced Defense Responses of Tomato Genotypes Differing in the JA Pathway
Source: PLoS One. 2011 May 24;6(5):e19751. doi: 10.1371/journal.pone.0019751 (PMC3101209; doi:10.1371/journal.pone.0019751)
Supplement: Table S3 — P values from MANOVAs for the effect of CO2 level, tomato genotype, and nematode infection on plant volatiles. (DOC) [file pone.0019751.s003.doc]

**Table S3.** *P* values from MANOVAs for the effect of CO_2_ level, tomato genotype, and nematode infection on plant volatiles.

| Volatiles | CO_2_^a^ | Genotype^b^ | Nematode^c^ | CO_2_×Genotype | CO_2_×Nematode | Genotype×Nematode | CO_2_×Genotype×Nematode |
| --- | --- | --- | --- | --- | --- | --- | --- |
| (E)-2-hexenal | 0.838 | <0.001*** | <0.001*** | 0.176 | 0.241 | 0.004 ** | 0.152 |
| *α*-pinene | 0.051 | <0.001*** | 0.001 ** | 0.867 | 0.158 | 0.035 * | 0.774 |
| *β*-pinene | 0.634 | <0.001*** | <0.001*** | 0.842 | 0.384 | 0.119 | 0.772 |
| carene | 0.038 * | <0.001*** | 0.001 ** | 0.166 | 0.104 | 0.296 | 0.870 |
| *β*-myrcene | 0.182 | <0.001*** | 0.841 | 0.180 | 0.277 | 0.573 | 0.748 |
| *α*-phellandrene | 0.047 * | <0.001*** | 0.005 ** | 0.259 | 0.039 * | 0.361 | 0.780 |
| ocimene | 0.010 * | <0.001*** | 0.438 | 0.042 * | 0.075 | 0.044 * | 0.421 |
| limonene | 0.861 | <0.001*** | <0.001*** | 0.138 | 0.130 | 0.638 | 0.423 |
| *β*-phellandrene | 0.037 * | <0.001*** | <0.001*** | 0.032 * | 0.054 | 0.319 | 0.764 |
| Total release | 0.028 * | <0.001*** | 0.001 ** | 0.059 | 0.048 * | 0.359 | 0.798 |
| ^a^ Ambient CO_2_ vs. elevated CO_2_.  ^b^ Three genotypes of tomato (s*pr2*, Wt, and *35S::prosys*).  ^c^ 7 days post-inoculation or 14 days post-inoculation or not inoculated with the root-knot nematode *M. incognita*.  *<0.05, **<0.01, ***<0.001 | | | | | | | |
