# Supplementary material for: Elevated CO2 Influences Nematode-Induced Defense Responses of Tomato Genotypes Differing in the JA Pathway
Source: PLoS One. 2011 May 24;6(5):e19751. doi: 10.1371/journal.pone.0019751 (PMC3101209; doi:10.1371/journal.pone.0019751)
Supplement: Table S4 — Emission ratea of volatile organic compounds (VOC) from tomato genotypes grown under ambient (390 ppm) and elevated CO2 (750 ppm) without and with M. incognita. (DOC) [file pone.0019751.s004.doc]

**Table S4.** Emission rate^a^ of volatile organic compounds (VOC) from tomato genotypes grown under ambient (390 ppm) and elevated CO_2_ (750 ppm) without and with *M. incognita*.

| Genotype | Volatiles | 370 ppm | | | 750 ppm | | |
| --- | --- | --- | --- | --- | --- | --- | --- |
|  |  | -^b^ | 7 dpi^c^ | 14 dpi^d^ | - | 7 dpi | 14 dpi |
| *spr2* | (*E*)-2-hexenal | 2.83±0.61b,B | 2.86±0.10b,B | 4.29±0.42ab,B | 3.71±0.74ab,A | 3.38±0.56ab,A | 5.14±0.97a,B |
|  | *α*-pinene | 48.2±9.31a,B | 49.2±4.73a,A | 55.5±9.44a,B | 36.7±12.9a,A | 46.0±3.87a,B | 43.4±3.50a,B |
|  | *β*-pinene | 2.78±0.14b,A | 13.3±2.12a,A | 15.0±6.63a,B | 2.80±0.45b,B | 12.2±1.02a,A | 8.36±1.09a,B |
|  | 4-carene | 179.0±48.3a,AB | 71.1±35.3bc,A | 123.6±21.4ab,B | 73.9±20.7ab,A | 43.9±15.0c,B | 56.2±13.9bc,C |
|  | *β*-myrcene | 6.87±1.24a,B | 4.75±1.15ab,A | 5.94±1.15ab,B | 3.96±0.86ab,B | 3.63±0.61b,B | 2.95±0.30b,B |
|  | *α*-phellandrene | 20.2±4.43a,AB | 7.74±3.39b,A | 14.3±2.53ab,AB | 8.69±2.23ab,A | 6.41±1.65b,B | 6.55±1.33b,C |
|  | ocimene | 10.2±4.19a,AB | 5.41±1.66ab,A | 5.00±0.82ab,A | 2.57±0.76b,A | 2.85±0.31b,B | 2.65±0.67b,C |
|  | limonene | 53.4±6.64a,B | 15.9±5.77b,A | 27.0±8.29ab,B | 29.2±5.55ab,B | 16.6±4.29b,B | 13.5±4.08b,C |
|  | *β*-phellandrene | 237.0±45.4a,AB | 88.2±41.6c,A | 202.8±49.7ab,AB | 82.4±21.0bc,B | 62.5±24.5c,B | 71.7±15.7c,C |
| Wt | (*E*)-2-hexenal | 2.09±0.48d,B | 5.65±1.44bc,AB | 9.20±1.75ab,A | 2.01±0.19d,A | 4.77±1.23c,A | 11.4±2.32a,A |
|  | *α*-pinene | 45.3±7.49ab,B | 49.7±5.40ab,A | 70.5±4.92a,B | 35.6±7.12b,A | 50.1±7.46ab,B | 55.4±12.3a,B |
|  | *β*-pinene | 2.96±0.80b,A | 12.2±4.35a,A | 27.1±9.77a,B | 3.55±0.47b,B | 12.2±4.35a,A | 20.1±4.31a,A |
|  | 4-carene | 114.8±26.0a,B | 45.7±4.39b,A | 138.1±49.2a,B | 69.7±14.5ab,A | 45.7±4.39b,B | 134.1±20.3a,B |
|  | *β*-myrcene | 4.58±0.95a,B | 4.56±1.70a,A | 5.90±1.55a,B | 4.10±0.71a,B | 4.88±2.02a,B | 6.25±1.31a,A |
|  | *α*-phellandrene | 14.2±3.41a,B | 5.60±0.48b,A | 13.3±4.49a,B | 7.68±1.78ab,A | 5.60±0.48b,B | 13.8±2.75a,B |
|  | ocimene | 4.52±0.98ab,B | 5.46±2.40ab,A | 10.7±6.13a,A | 2.47±0.66ab,A | 2.23±1.30b,B | 6.44±0.74a,B |
|  | limonene | 38.0±8.15a,B | 18.7±7.20b,A | 22.1±4.10ab,B | 33.2±4.36ab,B | 18.7±7.20b,B | 34.4±5.03ab,B |
|  | *β*-phellandrene | 141.0±36.7ab,B | 57.5±7.72c,A | 173.8±55.8a,B | 79.7±15.5bc,B | 57.5±7.72c,B | 180.4±37.6a,B |
| *35S* | (*E*)-2-hexenal | 9.47±2.47a,A | 9.88±1.26a,A | 14.7±3.7a,A | 3.73±1.13b,A | 10.2±0.76a,A | 16.2±1.97a,A |
|  | *α*-pinene | 128.5±19.3ab,A | 64.3±7.14c,A | 146.4±21.3a,A | 78.0±17.4bc,A | 73.3±10.8c,A | 161.2±29.6a,A |
|  | *β*-pinene | 8.46±1.80b,A | 42.6±19.2a,A | 46.5±4.84a,A | 6.45±1.07b,A | 56.0±12.9a,A | 34.9±5.45a,A |
|  | 4-carene | 345.5±55.8a,A | 188.5±78.2a,A | 300.0±26.7a,A | 213.5±70.8a,A | 254.8±48.1a,A | 278.5±51.4a,A |
|  | *β*-myrcene | 13.3±2.33a,A | 13.4±6.58a,A | 14.6±1.19a,A | 10.9±2.68a,A | 18.6±4.48a,A | 10.4±2.01a,A |
|  | *α*-phellandrene | 39.9±7.38a,A | 23.0±10.3a,A | 29.2±2.16a,A | 25.1±8.41a,A | 32.9±8.05a,A | 26.8±4.61a,A |
|  | ocimene | 14.4±2.54ab,A | 16.8±3.83a,A | 7.14±0.96b,A | 8.64±3.23b,A | 17.5±3.45a,A | 21.5±4.47a,A |
|  | limonene | 137.3±16.2a,A | 62.2±32.7b,A | 58.7±5.32ab,A | 87.4±16.9ab,A | 87.6±21.6ab,A | 97.1±11.1ab,A |
|  | *β*-phellandrene | 464.5±69.8ab,A | 279.7±115.9b,A | 397.3±58.0ab,A | 289.0±66.5ab,A | 366.5±61.0ab,A | 502.2±52.1a,A |
| ^a^ Emission rate = ng of compound released by 10 g (fresh weight) of leaves per hour. ^b^ not inoculated with *M. incognita*. ^c^ 7 days post-inoculation. ^d^ 14 days post-inoculation. Each value represents the average (±SE) of 3 replicates. Different lowercase letters within a row indicate significant differences (LSD test: d.f.=5, 12; *P <*0.05); Different uppercase letters indicate significant differences among tomato genotypes within the same CO_2_ and nematode treatment (LSD test: d.f.=2, 6; *P <*0.05). | | | | | | | |
