# Supplementary material for: Elevated CO2 Influences Nematode-Induced Defense Responses of Tomato Genotypes Differing in the JA Pathway
Source: PLoS One. 2011 May 24;6(5):e19751. doi: 10.1371/journal.pone.0019751 (PMC3101209; doi:10.1371/journal.pone.0019751)
Supplement: Table S5 — Primer sequences used in the real-time quantitative PCR. (DOC) [file pone.0019751.s005.doc]

**Table S5.** Primer sequences used in the real-time quantitative PCR.

| Gene | Primer sequence（5’-3’） | Fragment length (bp) | Function |
| --- | --- | --- | --- |
| *PI* | *F*: GCC AGA ACT TAT TGG TGT A  *R*: TGA CAT ATT GTG GCT GCT T | 230 | JA defense related |
| *LOX* | *F*: ATG GCG ACA AGA AAG ATG AGG  *R*: CTT AAA GTA GGG CGA TTA GGG | 163 | JA defense related |
| *PAL* | *F*: AGA TTG AAG TCA TTC GTG CTG  *R*: ACC CGT TGT TGT AAT AGT CGT TG | 228 | Secondary metabolites |
| *RUBISCO* | *F*: GGC TTC CTC TGT CAT TTC TTC  *R*: TGC AGC TAA CTC TTC CAC CAT | 170 | Photosynthesis related |
| *GST* | *F*: GCA CAT TTC AAA GCT CGT T  *R*: AAC CCA CAT GCA CTC ATA AAC | 151 | Active oxygen related |
| *PR* | *F*: TAC GCT ACC AAC CAA TGT G  *R*: TCC AGT TGC CTA CAG GAT C | 151 | SA defense related |
| *BGL* | *F*: ATC TTG AAG CCC TAG CCA ATC  *R*: GGA CCA ACA AAT CGT GCG TAT | 154 | SA defense related |
| *β-actin* | *F*: GTT GGA ATG GGT CAG AAA GAT  *R*: TTC AGT AAG CAG AAC AGG GTG | 189 | Housekeeping gene |
